# Artificial Intelligence Across the Echocardiographic Workflow: A Narrative Review for Clinicians

**DOI:** 10.7759/cureus.112828

**Published:** 2026-07-16

**Authors:** Dominik Skoczylas, Katarzyna Deleska, Wiktoria Chmura, Dawid M Buczynski, Anna Rog, Natalia Kwiatkowska, Lukasz Skoczylas, Urszula Klimczak

**Affiliations:** 1 Faculty of Medicine, Jagiellonian University Medical College, Kraków, POL; 2 Students' Scientific Group at the 1st Department of Cardiology, Interventional Electrocardiology and Arterial Hypertension, Jagiellonian University Medical College, Kraków, POL; 3 Radiology, Luxmed, Wroclaw, POL; 4 Faculty of Electrical Engineering, Automatics, Computer Science and Biomedical Engineering, Akademia Górniczo-Hutnicza (AGH) University of Kraków, Kraków, POL

**Keywords:** artificial intelligence in medicine, automated segmentation, clinical effectiveness, deep learning artificial intelligence, echo cardiogram, explainable ai (xai)

## Abstract

Echocardiography is one of the most commonly used diagnostic methods in cardiovascular diseases because it is non-invasive, widely available, and capable of providing real-time assessment of function and cardiac structure. Despite these advantages, conventional echocardiography is often limited by operator dependence, interobserver inconstancy, the time-intensive nature of manual acquisition and measurement. Recent advances in artificial intelligence (AI), particularly deep learning, have created new opportunities to automate multiple steps of the echocardiographic workflow, starting from image acquisition and view classification to chamber segmentation, functional quantification, and hemodynamic estimation.

This review provides an overview of the current clinical applications of artificial intelligence (AI) throughout the echocardiographic workflow. It focuses on key areas where AI has been applied, including automated view recognition, image quality assessment, cardiac phase identification, chamber segmentation, left ventricular ejection fraction estimation, strain analysis, and prediction of hemodynamic and disease-related parameters. Major challenges limiting wider clinical implementation were also highlighted in this review, such as insufficient external validation, dependence on image quality, differences between ultrasound vendors and patient populations, limited model interpretability, and lack of clear evidence demonstrating improved patient outcomes.

Several AI-based tools, particularly those for automated view classification and chamber quantification, are becoming increasingly integrated into routine clinical practice, but many more advanced applications are possible, for which research is ongoing. The successful adoption of AI in echocardiography will depend not only on continued improvements in algorithm performance, but also on rigorous clinical validation, smooth integration into existing workflows, transparent reporting of model development and evaluation, and evidence that these technologies provide meaningful benefits for patient care.

## Introduction and background

Echocardiography is one of the most common and noninvasive diagnostic tools that allows to diagnose and manage different cardiovascular diseases. It is a first-line imaging modality in a wide range of clinical settings, including outpatient practice, emergency medicine, intensive care, and perioperative care.

Despite its clinical importance, conventional echocardiography has several limitations. The final outcome depends heavily on operator experience, may vary between readers, and is time-consuming when performed manually [1]. The assessment of left ventricular ejection fraction (LVEF), chamber dimensions, valvular function, and deformation parameters may therefore be affected both by technical and human factors [1].

The usage of artificial intelligence (AI) as a tool can overcome these limitations. AI-driven systems allow for maximizing reproducibility by automating image acquisition, measurement, and interpretation. In this way, they can minimize inter- and intra-observer variability [2,3]. Automating repetitive tasks can also improve workflow efficiency such as quantification of LVEF and cardiac view recognition, which reduces the time required for manual measurements [3]. When it comes to operator dependency and their experience in interpretation, AI-guided probe positioning combined with real-time feedback can help less experienced clinicians to acquire high-quality images. AI performance is rapidly getting more and more accurate in detecting subtle abnormalities, for example, in detecting myocardial texture changes accurately is comparable to expert cardiologists [3,4]. This could allow novice operators to gain reliable results and to make echocardiography more accessible in underserved areas.

Deep learning algorithms (DL) have significantly transformed the field of medical imaging in echocardiography, compared to traditional image analysis. DL algorithms, such as convolutional neural networks (CNNs) and recurrent neural networks (RNNs), have revolutionized image analysis by automating classification, segmentation, and disease diagnosis [5]. These methods have demonstrated higher accuracy and efficiency when compared to traditional techniques [6]. In addition, the integration of DL algorithms into portable handheld echocardiography devices can make high-quality cardiac care more accessible [7].

This article is structured as a narrative review focused on clinically relevant usage of AI in echocardiography. The literature that was selected to be studied followed principles such as: the latest database and publication year - prioritizing recent years; key words such as clinical implementation, clinical practice, practical approach; the most representative database, meaning comparing the obtained results with currently existing tools and programmes; addressing key stages of the echocardiographic workflow, including image acquisition, view classification, quality assessment, cardiac phase identification, segmentation, functional quantification, deformation imaging, hemodynamic estimation, and interpretability.

## Review

View classification and quality assessment

To understand the role of AI in echocardiography, we need to think about it as a sequence of applications integrated across the imaging pipeline. Before clinically meaningful measurements can be generated, several upstream tasks must be completed reliably. These include acquisition of a diagnostically adequate study, recognition of the imaging plane, identification of the relevant phase of the cardiac cycle, and exclusion of images that are unsuitable for analysis. Only after these preparatory steps are done, tasks such as chamber segmentation, quantification of ventricular function, deformation analysis, and hemodynamic interpretation can be performed with confidence.

Automatic view classification is the natural first step. A transthoracic study consists of more than a dozen standard planes, among them the apical two- and four-chamber views (2CH, 4CH) and the parasternal long- and short-axis views (PLAX, PSAX), and the algorithm must tell them apart before it can apply the appropriate measurement rules to a given image. Early work by Gao et al. extended convolutional networks to this task by fusing two streams: one encoding spatial appearance and the other temporal motion derived from dense optical flow, reaching 92.1% accuracy across eight viewpoints and showing that motion information enhanced the performance substantially over still frames alone [8]. The landmark study by Madani et al. then trained a single CNN to recognize 15 standard views simultaneously, achieving 97.8% accuracy on video clips and roughly 92% on isolated low-resolution frames, clearly exceeding the agreement obtained by experienced echocardiographers on the same material [4]. The problem soon shifted from feasibility to deployment: Østvik et al. demonstrated real-time view classification running live during acquisition [9], while in the fully automated pipeline of Zhang et al., a module identifying 23 viewpoints served as the entry stage feeding chamber segmentation, functional quantification, and disease detection on a corpus of 14,035 echocardiograms collected over a decade [10].

The second step is identifying the phases of the cardiac cycle, pinpointing the end-diastolic (ED) and end-systolic (ES) frames. Conventionally, these instants are read off a synchronous ECG trace, but such a signal is not always available or reliable, and its absence should not stall the analysis. Taheri Dezaki et al. cast the problem as regression, combining image feature extractors (DenseNet, ResNet) with recurrent networks that model the temporal dependencies between consecutive frames (long short-term memory (LSTM), Bi-LSTM, gated recurrent unit (GRU), Bi-GRU), trained under a purpose-built global-extrema loss [11]. Lane et al. pushed this towards clinical practice with a model that handles multibeat cine loops of arbitrary length and was validated against annotations from seven cardiologists across multiple centres, reporting frame errors close to expert interobserver variability [12]. The same logic of working straight from the image stream underpins EchoNet-Dynamic, which operates across several consecutive heartbeats, segments the left ventricle with a Dice coefficient of 0.92, and estimates ejection fraction with a mean absolute error of about 4.1% in a genuine beat-to-beat fashion [13].

The third step is gating and quality assessment. Echocardiography is exceptionally operator-dependent and prone to artifacts such as acoustic shadowing, reverberation, and a low signal-to-noise ratio, and a measurement taken from a poor image can be worse than no measurement at all. Earlier approaches inspected the signal spectrum and were essentially blind to anatomy: they could flag obstruction but could not judge whether the correct cardiac structures were actually visible. The shift came with Abdi et al., whose CNN scored the quality of the apical four-chamber view against a set of clinical criteria and produced an objective quality index intended to support the operator during acquisition itself [14]. Related acquisition-quality work by Smistad et al. detected and quantified apical foreshortening, which is a common scanning error that biases volume and ejection-fraction estimates, directly within a real-time measurement pipeline [15]. Benchmarking efforts have reinforced the same priority: the publicly available Cardiac Acquisition for Multi-structure Ultrasound Segmentation (CAMUS) dataset assembled by Leclerc et al. annotates each acquisition by image quality, enabling models to be trained and tested with explicit awareness of how degraded inputs affect downstream segmentation and volume estimation [16]. Modules of this kind reject non-diagnostic recordings at the beginning of a pipeline. Without them, even the best measurement model would be operating on data a human reader would never have accepted.

Automated segmentation

Clinical Importance of Segmentation

Automated segmentation is the foundation of modern echocardiography methodology. It is crucial that the structures are accurately measured so as to avoid subjectivity and human error. In clinical terms, its value lies not in replacing clinical judgement, but in reducing the burden and variability of repetitive measurements. Among all AI applications in echocardiography, chamber segmentation and automated LVEF calculation are the most popular, already in use.

The foundation of the field of deep learning architectures is U-Net. It connects high-resolution details with broader structural information by “skip connections” of its unique U-shaped design. It is very effective at ensuring that any detail, for example, thin cardiac walls, is not overlooked. However, a common struggle for standard U-Net can be images with a lot of artifacts and high levels of noise [17,18].

To solve problems encountered while using U-Net, Attention U-Net has been created. This new model includes "attention gates” that act as a filter that ignores artifacts and background noises of ultrasound images and prioritizes relevant cardiac tissue. This improves accuracy as the model becomes less sensitive to poor-quality imaging and also by emphasizing clinically relevant regions [6,18].

Furthermore, TransUNet has been developed as a hybrid model that combines the advantages of two technologies: CNNs and Transformers. The fact that it uses subtle local details such as fine borders of CNNs and Transformer’s awareness of the entire heart anatomy makes this model versatile and provides a holistic approach in turning automated segmentation into a reliable tool for high-precision hemodynamic assessment [19]. The main features of different deep learning architectures are summarized in Table 1.

**Table 1:** Comparison of Deep Learning Architectures for Echocardiographic Segmentation CNN: Convoluted neural network; LA: left atria; LV: left ventricle. Sources: Refs. [6,17-19].

Architecture	Primary Strength	Mechanism for Improvement	Clinical Best Use-Case
U-Net	Spatial detail preservation	Skip connections	Standard, clean images; baseline tasks.
Attention U-Net	Noise suppression	Attention gates	Noisy images; improving measurement reproducibility.
TransUNet	Holistic (global) context	Hybrid CNN-Transformer design	Complex structures (LA, RV); high-precision analysis.

Two-dimensional versus Three-dimensional Analysis

Because our heart naturally is not 2D, this method of segmentation is limited. It relies on geometric structures and is prone to subjective interpretation, which can lead to inaccuracies in ejection fraction and stroke volume calculations. It often overlooks the full, dynamic reality of the beating heart [20-22].

For true physiological assessment, it is crucial to move toward 3D and 4D (3D+t) segmentation. The “t” represents time, as 4D and 3D+t models represent the full heart cycle. The main technical challenge is ensuring spatio-temporal consistency [23], thus providing a full and smooth moving image representing smooth and anatomically realistic models of the moving human heart, rather than a “jittery” or disconnected one. These models use advanced temporal tracking to smooth out noise and artifacts to obtain far more reliable volumetric data, providing objective, high-precision measurements needed to reflect a patient's true hemodynamic status.

Left Ventricular Versus Right Ventricular Segmentation

Automated cardiac segmentation is technical because there are significant differences among the heart ventricles. While the left ventricle (LV) serves as the benchmark for model performance with its relatively regular geometry compared with other chambers; segmenting the right ventricle (RV) and atria (LA/RA) can be challenging. The RV’s irregular, crescent-like shape and the atria’s thin, compliant walls often result in blurred or indistinct boundaries in standard echocardiographic images [24,25]. To get around these hurdles, the latest generation of AI has moved toward "hybrid" models, which combine the strengths of standard convolutional networks with Transformer-based architectures. Thus, it uses global context to understand the shape of the entire heart [19,25]. By "zooming out" to see the whole anatomy while simultaneously sharpening its focus on thin-walled structures, these models provide consistent, reliable measurements that clinicians actually need for daily practice.

Current limitations of segmentation-based automation

Several important limitations remain. First, model performance is strongly dependent on input image quality. Second, automated segmentation may not generalize equally across vendors, populations, and acquisition protocols. However, deep learning models, such as U-Net and Transformer-based architectures, have shown promising results in improving segmentation accuracy, even in low-contrast or artifact-laden echocardiographic images [6,22].

The Simpson’s biplane method is the conventional approach for calculating LV end-diastolic volume (EDV), end-systolic volume (ESV), and ejection fraction (LVEF), and automated AI applications have been introduced to reproduce it [26]. To automate these calculations, AI-based tools, such as convolutional neural networks, have been developed. These tools demonstrate strong correlations with manual methods, with reduced variability and improved reproducibility [22,26]. Fully automated algorithms, such as those using 3D echocardiography and other tomographic modalities (cardiac CT, single-photon emission computerized tomography (SPECT), or MRI), provide accurate volumetric measurements and LVEF calculations, often outperforming traditional 2D methods in patients with complex conditions like regional dyssynchrony [27-29].

Emerging Solutions

AI models, such as Bi-GAN (generative adversarial network) and EchoSAM, are being developed to address challenges like low contrast and noise in echocardiographic images. These models have demonstrated high accuracy in segmenting cardiac structures and calculating functional parameters [22].

Advanced functional assessment: strain and motion analysis

Clinical Relevance of Strain Imaging

Myocardial deformation imaging provides information that is complementary to conventional measures such as ejection fraction. Global longitudinal strain (GLS) is an important marker of subclinical left ventricular dysfunction and has clinical relevance in cardio-oncology, valvular disease, ischemic heart disease, and heart failure [30,31]. However, strain analysis is often time-consuming, vendor-dependent, and sensitive to image quality. That is why AI-supported or AI-automated approaches can be very useful and can standardize tracking and reduce manual burden.

In the context of cardio-oncological care, automated GLS evaluation plays a key role in long-term patient monitoring. It enables the identification of subtle, potentially reversible contractility abnormalities at a stage when the implementation of appropriate cardioprotection allows for the preservation of the heart’s functional integrity [32,33]. The implementation of advanced deep learning (DL) algorithms in automated GLS systems has led to a significant improvement in the reproducibility of results. By eliminating the need for manual tracing of endocardial contours, these technologies drastically reduce human unsteadiness. As a result, automated GLS has become an objective, standardized tool dedicated to early cardiovascular risk stratification in everyday clinical practice [34].

AI-Assisted Speckle Tracking and Motion Analysis

Three-dimensional speckle-tracking echocardiography (3D STE) has the ability to assess area strain, which is characterized by excellent reproducibility, unique sensitivity, and specificity in mapping regional differences of contraction [35,36]. The clinical utility of this method has been compared in comparative studies with cardiac magnetic resonance (CMR) and, as a result, a high spatial correlation was observed between strain maps and the presence of post-infarction scarring in late gadolinium enhancement (LGE), particularly in the case of transmural infarcts [37].

From a clinical perspective, the value of these methods lies in improving reproducibility and potentially making advanced functional assessment more accessible in routine practice. Automated GLS estimation may be particularly attractive because GLS often detects subtle systolic dysfunction earlier than LVEF. This is relevant in patients receiving potentially cardiotoxic therapies, in early cardiomyopathy, and in selected valvular and ischemic conditions.

Hemodynamic estimation and predictive modeling

A major frontier in AI echocardiography is the extension of automated analysis from structural quantification to physiological interpretation. Beyond measuring chamber size and systolic function, AI models are increasingly being developed to estimate filling pressures, characterize valvular lesions, classify disease phenotypes, and predict clinically important outcomes [38,39].

This shift is important because the ultimate clinical role of echocardiography is not merely image description but physiological and diagnostic interpretation. If AI can contribute meaningfully to hemodynamic assessment, its value may extend well beyond measurement automation.

Hemodynamic Estimation

Several studies have explored AI-based estimation of hemodynamic variables using standard echocardiographic data. Potential applications include the estimation of left ventricular filling pressures, support for diastolic function assessment, and integration of Doppler and structural parameters into clinically meaningful classifications. These approaches may be especially useful in situations where conventional interpretation is complex or subject to substantial interobserver variability [40-42].

Still, hemodynamic estimation is considerably more challenging than tasks such as view recognition or left ventricular segmentation. Many physiological parameters are context-dependent, influenced by loading conditions, rhythm, heart rate, and comorbidity.

Integration of Virtual Echocardiography with Computational Fluid Dynamics (CFD)

The synergy between automated three-dimensional time-resolved geometry segmentation (3D+t) and CFD has enabled "virtual echocardiography," providing a granular, patient-specific window into intracardiac fluid mechanics [43]. While traditional imaging struggles to map transient spatial shear forces, automated deep learning architectures (such as 3D nnU-Net variants) generate fluid-velocity-ready meshes with rapid inference speeds directly from volumetric cardiac imaging data [43].

Advanced modalities like volumetric ultrasound image velocimetry (VUIV), vector flow imaging (VFI), and echo particle image velocimetry (Echo PIV) complement these CFD frameworks, offering superior measurement alignment against reference-standard cardiac magnetic resonance imaging [44,45]. Crucially, this integration moves diagnostic capabilities beyond basic velocity vectors, allowing clinicians to quantify abstract biomechanical parameters including energy loss (EL), energy dissipation across cardiac cycles, and turbulent kinetic energy (TKE) [46]. These combined metrics expose regional turbulence intensities and structural workloads, linking morphological tracking directly with the underlying vascular friction profiles [46,47].

AI-Driven Prediction of Hemodynamic Reserves and Therapeutic Response

Furthermore, multimodal deep learning frameworks are changing patient selection for cardiac resynchronization therapy (CRT). By synthesizing diverse inputs, including localized myocardial strain profiles, electrocardiographic parameters, and clinical biomarkers, the hybrid models can reach up to 85% accuracy in predicting therapeutic response, far exceeding the ~70% accuracy of traditional guideline criteria [48,49]. Unsupervised machine learning models assist this process by phenotyping patients into different clinical clusters based on mechanical dyssynchrony and regional time-to-peak deformation profiles [48]. Driven by explainable AI modules (such as Shapley additive explanations (SHAP), local interpretable model-agnostic explanations (LIME), and gradient-weighted class activation mapping (Grad-CAM)) and uncertainty-aware calibration, these systems visually map the underlying logic behind their predictive thresholds [50,51]. This provides clinicians with transparent, multi-stage decision pathways that optimize pacing configurations, predict hemodynamic reserves, and mitigate the risks of therapeutic non-response [52].

Clinical credibility and explainable artificial intelligence (XAI)

The "Black Box" Problem

Deep learning models tend to lack transparency and interpretability, which create an understandable clinician resistance to AI-generated diagnoses. It is vital to understand and evaluate the model’s decision-making process to be able to use them in clinical practice in echocardiography [3,53,54]. Furthermore, algorithmic bias should be taken into consideration. A reliable model has to be trained on a diverse dataset including images from different ultrasonographs, acquired by distinct specialists on different populations. This approach allows the model to be more precise, generalizable, and robust, which can increase the quality of diagnosis and therefore clinical trust [3,7,53]. Explainable AI (XAI) comes as a solution with techniques like Grad-CAM, SHAP, and LIME, but their application in echocardiography still remains limited [7,55].

Explainable AI (XAI) methods in echocardiography

Grad-CAM

This method highlights the regions of the heart that are crucial to the model's predicted diagnosis such as valves and abnormal flow jets [56]. A heatmap explaining the model’s focus is generated, which allows the clinicians to understand the base of decision-making [56].

Saliency Maps

For disease classification tasks (identifying myocardial abnormalities or valvular diseases), saliency maps can be used because they visualize the most critical areas in echocardiographic images [55]

Integrated Gradients

Integrated gradients (IG) quantify the exact contribution of individual pixels to the model’s output. This approach is far more detailed compared to Grad-CAM. Moreover, IG is highly capable of matching clinical experts’ analysis, making it a reliable tool for clinicians [55].

Attention Mechanisms

Echocardiography images are often noisy and low-contrast, therefore making the diagnosis more challenging. Attention mechanisms provide a solution to this problem. Attention-guided Grad-CAM has been developed to address these issues as they restrict visual explanations to the most deterministic anatomical regions [55]. Furthermore, such models can accurately trace the boundaries of cardiac structures, improving the clinical validity [56].

Why Explainability Alone Is Not Enough

Although explainability is valuable, it should not be mistaken for validation. A visually plausible heatmap does not prove that a model is robust, unbiased, or clinically reliable. True clinical credibility requires a broader evidence base that includes external validation, calibration, and performance across multiple vendors and institutions, and testing in real-world workflows.

For this reason, interpretability should be understood as a supportive feature rather than a substitute for methodological rigor. A clinically credible AI system is one that is transparent enough to be reviewed, robust enough to perform consistently, and well validated enough to be trusted in practice.

Generalizability and Bias

Generalizability remains one of the most serious unresolved issues in AI echocardiography. Many models are trained on single-center data, relatively homogeneous populations, or carefully curated images that do not reflect the full variability of real-world practice. Differences in ultrasound vendors, transducer settings, acquisition protocols, patient body habitus, and disease prevalence may all influence model performance [57,58].

Bias may also arise if training datasets inadequately represent women, older adults, minority populations, patients with poor acoustic windows, or those with uncommon pathology. A model that performs well overall may still perform inconsistently in clinically important subgroups. These concerns are not merely technical; they directly affect fairness, safety, and the reliability of AI-assisted care.

Clinical application of AI tools in practice 

The main clinical problems that can be encountered in daily practice and the most suitable AI approach are summarized in Table 2.

**Table 2:** Summary of AI Approaches for Key Clinical Problems in Echocardiography CNN: Convolutional neural networks; LVEF: left ventricular ejection fraction; GLS: global longitudinal strain; RNN: recurrent neural networks; LSTM: long short-term memory; CFD: computational fluid dynamics. Source: Refs. [4,6,11,17-19,30,31,40-42,46-52].

Clinical problem	Most suitable AI approach
View recognition and scan quality	CNN-based classification and quality-assessment models
LVEF, chamber volumes, routine segmentation	U-Net, Attention U-Net, TransUNet
Early myocardial dysfunction	Deep learning-enhanced GLS and automated speckle tracking
Regional ischemia and infarct localization	Optical flow models, RNN/LSTM, 3D strain mapping
Diastolic dysfunction and filling pressures	ML models integrating Doppler and atrial strain data
Advanced flow and structural hemodynamics	3D segmentation plus CFD-based modeling
Prediction of therapeutic response	Multimodal deep learning models
Clinician trust and implementation	Explainable AI methods

In routine transthoracic echocardiography, CNNs remain the most effective tools for view classification, quality assessment, and basic chamber recognition.

For LVEF, chamber volumes, and routine segmentation, U-Net-derived architectures remain the most clinically useful tools. Standard U-Net performs well in relatively clean and conventional studies, while Attention U-Net is more suitable when image quality is limited by noise, reverberations, or poor acoustic windows. TransUNet and other hybrid CNN-Transformer models appear most promising for more anatomically difficult structures such as the right ventricle and atria.

For subclinical myocardial dysfunction, especially in patients whose LVEF is still preserved, the most useful AI-supported tool is automated global longitudinal strain (GLS) derived from deep learning-enhanced speckle tracking echocardiography. This appears particularly valuable in cardio-oncology, where AI-assisted GLS can identify early anthracycline-related myocardial injury before conventional ejection fraction changes become visible.

For regional wall motion abnormalities, ischemia detection, and infarct localization, the strongest tools are optical-flow-based models, recurrent neural networks (RNNs/LSTMs), and 3D speckle-tracking systems, especially when combined with bull's-eye strain mapping. These models are particularly useful when the clinical question is not simply whether systolic function is reduced, but where the dysfunction is located and whether it reflects ischemia, scar, or dyssynchrony.

For hemodynamic assessment and filling pressure estimation, the most promising tools are machine learning models that integrate Doppler parameters with atrial strain and dynamic chamber mechanics, rather than relying only on traditional rule-based algorithms.

For advanced biomechanical modeling and patient-specific flow analysis, the most suitable tools are 3D segmentation networks integrated with CFD. For predicting response to therapy, especially cardiac resynchronization therapy (CRT), multimodal deep learning models appear to be the most clinically impactful tools currently emerging.

Finally, for clinical trust and adoption, the most important enabling tools are not diagnostic models themselves, but explainable AI techniques such as Grad-CAM, saliency maps, integrated gradients, SHAP, and LIME. Their greatest value lies in making AI clinically discussable. Physicians are far more likely to trust a system that highlights why it labeled a segment abnormal, why it considered one chamber border reliable, or why it predicted poor CRT response.

## Conclusions

Artificial intelligence is reshaping echocardiography across the full imaging pipeline, from acquisition support and view classification to chamber segmentation, functional quantification, and hemodynamic interpretation. Among these applications, automated view recognition, quality control, and left ventricular quantification are already said to be the closest to be used daily in clinical routine, especially because they can improve workflow efficiency and reduce measurement variability.

The most important lesson from the current literature is that artificial intelligence should not be viewed as a single technology. Its clinical impact depends on matching the right model to the right question.

Artificial intelligence is redefining echocardiography from a highly operator-dependent imaging modality into a more standardized, reproducible, and clinically scalable tool. Its value is greatest when applied to specific problems: improving view acquisition, automating segmentation, detecting early myocardial dysfunction, etc. For clinicians, the most important takeaway is that AI does not merely make echocardiography faster. It has the potential to make it more objective, more accessible, and more informative.

## References

[1] Gonzalez FA, Zawadka M, Varudo R (2025). Automated and reference methods for the calculation of left ventricular outflow tract velocity time integral or ejection fraction by non-cardiologists: a systematic review on the agreement of the two methods. J Clin Monit Comput.

[2] Ferraz S, Coimbra M, Pedrosa J (2023). Assisted probe guidance in cardiac ultrasound: a review. Front Cardiovasc Med.

[3] Sakamoto A, Kaneko T, Sato E, Fujita W, Nakamura Y, Yokotsuka N, Kagiyama N (2025). Artificial intelligence in echocardiography: current applications and future perspectives. J Echocardiogr.

[4] Madani A, Arnaout R, Mofrad M, Arnaout R (2018). Fast and accurate view classification of echocardiograms using deep learning. NPJ Digit Med.

[5] Zeng J, Zhang H, Liu H (2023). Research status of cardiac image segmentation based on deep learning. Journal of Image and Graphics.

[6] Chen C, Qin C, Qiu H, Tarroni G, Duan J, Bai W, Rueckert D (2020). Deep learning for cardiac image segmentation: a review. Front Cardiovasc Med.

[7] East SA, Wang Y, Yanamala N, Maganti K, Sengupta PP (2025). Artificial intelligence-enabled point-of-care echocardiography: bringing precision imaging to the bedside. Curr Atheroscler Rep.

[8] Gao X, Li W, Loomes M, Wang L (2017). A fused deep learning architecture for viewpoint classification of echocardiography. Information Fusion.

[9] Østvik A, Smistad E, Aase SA, Haugen BO, Lovstakken L (2019). Real-time standard view classification in transthoracic echocardiography using convolutional neural networks. Ultrasound Med Biol.

[10] Zhang J, Gajjala S, Agrawal P (2018). Fully automated echocardiogram interpretation in clinical practice. Circulation.

[11] Taheri Dezaki F, Liao Z, Luong C (2019). Cardiac phase detection in echocardiograms with densely gated recurrent neural networks and global extrema loss. IEEE Trans Med Imaging.

[12] Lane ES, Azarmehr N, Jevsikov J (2021). Multibeat echocardiographic phase detection using deep neural networks. Comput Biol Med.

[13] Ouyang D, He B, Ghorbani A (2020). Video-based AI for beat-to-beat assessment of cardiac function. Nature.

[14] Abdi AH, Luong C, Tsang T (2017). Automatic quality assessment of echocardiograms using convolutional neural networks: feasibility on the apical four-chamber view. IEEE Trans Med Imaging.

[15] Smistad E, Ostvik A, Salte IM (2020). Real-time automatic ejection fraction and foreshortening detection using deep learning. IEEE Trans Ultrason Ferroelectr Freq Control.

[16] Leclerc S, Smistad E, Pedrosa J (2019). Deep learning for segmentation using an open large-scale dataset in 2D echocardiography. IEEE Trans Med Imaging.

[17] Ignacio MJ, Shin S, Jin H, Yoo SJ, Han D, Kim YG (2025). Revisiting U-Net: a foundational backbone for modern generative AI. Artificial Intelligence Review.

[18] Hossen MF, Sarkar A, Majumder T (2026). A comprehensive review of U-Net architectures for medical image segmentation: emerging trends and federated learning perspectives. Neural Computing and Applications.

[19] Aghapanah H, Rasti R, Kermani S (2024). CardSegNet: an adaptive hybrid CNN-vision transformer model for heart region segmentation in cardiac MRI. Comput Med Imaging Graph.

[20] Dorosz JL, Lezotte DC, Weitzenkamp DA, Allen LA, Salcedo EE (2012). Performance of 3-dimensional echocardiography in measuring left ventricular volumes and ejection fraction: a systematic review and meta-analysis. J Am Coll Cardiol.

[21] Zhao D, Ferdian E, Maso Talou GD (2022). MITEA: a dataset for machine learning segmentation of the left ventricle in 3D echocardiography using subject-specific labels from cardiac magnetic resonance imaging. Front Cardiovasc Med.

[22] Li X, Hu Q, Lin X, Li Y, Dong Y, Lin T (2025). EchoSAM: SAM adaption for unified 2D echocardiography segmentation and ejection fraction calculation. Biomedical Signal Processing and Control.

[23] Lu W, Wang Y, Dai W, Wu Y, Xu H, Kong D (2025). Echo-ODE: a dynamics modeling network with neural ODE for temporally consistent segmentation of video echocardiograms. Front Physiol.

[24] Guo Z, Zhang Y, Qiu Z (2023). An improved contrastive learning network for semi-supervised multi-structure segmentation in echocardiography. Front Cardiovasc Med.

[25] Su C, Ma J, Zhou Y, Li P, Tang Z (2023). Res-DUnet: a small-region attentioned model for cardiac MRI-based right ventricular segmentation. Applied Soft Computing.

[26] Sveric KM, Botan R, Dindane Z (2023). Single-site experience with an automated artificial intelligence application for left ventricular ejection fraction measurement in echocardiography. Diagnostics (Basel).

[27] Kasai T, Depuey EG, Shah AA (2004). Compared with 3-dimensional analysis, 2-dimensional gated SPECT analysis overestimates left ventricular ejection fraction in patients with regional dyssynchrony. J Nucl Cardiol.

[28] Ghersin E, Abadi S, Yalonetsky S, Engel A, Lessick J (2009). Clinical evaluation of a fully automated model-based algorithm to calculate left ventricular volumes and ejection fraction using multidetector computed tomography. Acute Card Care.

[29] Ren B, Vletter WB, McGhie J, Soliman OI, Geleijnse ML (2014). Single-beat real-time three-dimensional echocardiographic automated contour detection for quantification of left ventricular volumes and systolic function. Int J Cardiovasc Imaging.

[30] Abramikas Ž, Jasiukevičiūtė I, Balčiūnaitė G, Glaveckaitė S, Palionis D, Valevičienė N (2025). Artificial intelligence performance in cardiac magnetic resonance strain analysis for aortic stenosis: validation with echocardiography and healthy controls. Medicina (Kaunas).

[31] Rogstadkjernet M, Zha SZ, Klæboe LG (2024). A deep learning based method for left ventricular strain measurements: repeatability and accuracy compared to experienced echocardiographers. BMC Med Imaging.

[32] Mele D, Malagutti P, Indelli M (2016). Reversibility of left ventricle longitudinal strain alterations induced by adjuvant therapy in early breast cancer patients. Ultrasound Med Biol.

[33] Chang HY, Lee CH, Su PL (2021). Subtle cardiac dysfunction in lymphoma patients receiving low to moderate dose chemotherapy. Sci Rep.

[34] Myhre PL, Hung CL, Frost MJ (2024). External validation of a deep learning algorithm for automated echocardiographic strain measurements. Eur Heart J Digit Health.

[35] Kleijn SA, Aly MF, Terwee CB, van Rossum AC, Kamp O (2011). Three-dimensional speckle tracking echocardiography for automatic assessment of global and regional left ventricular function based on area strain. J Am Soc Echocardiogr.

[36] Wen H, Liang Z, Zhao Y, Yang K (2011). Feasibility of detecting early left ventricular systolic dysfunction using global area strain: a novel index derived from three-dimensional speckle-tracking echocardiography. Eur J Echocardiogr.

[37] Kylmälä MM, Antila MK, Kivistö SM (2008). Tissue Doppler strain-mapping in the assessment of the extent of chronic myocardial infarction: validation using magnetic resonance imaging. Eur J Echocardiogr.

[38] Nedadur R, Wang B, Tsang W (2022). Artificial intelligence for the echocardiographic assessment of valvular heart disease. Heart.

[39] Maznyczka A, Nuis RJ, Shiri I (2025). Artificial intelligence in valvular heart disease: innovations and future directions. JACC Cardiovasc Interv.

[40] Park J, Jeon J, Yoon YE (2024). Artificial intelligence-enhanced automation of left ventricular diastolic assessment: a pilot study for feasibility, diagnostic validation, and outcome prediction. Cardiovasc Diagn Ther.

[41] Karužas A, Miščikas L, Kiziela A (2025). Deep learning-driven automated assessment of left ventricular diastolic function in echocardiography. Echocardiography.

[42] Qayyum SN (2024). A comprehensive review of applications of artificial intelligence in echocardiography. Curr Probl Cardiol.

[43] Yao T, Pajaziti E, Quail M, Schievano S, Steeden J, Muthurangu V (2024). Image2Flow: a proof-of-concept hybrid image and graph convolutional neural network for rapid patient-specific pulmonary artery segmentation and CFD flow field calculation from 3D cardiac MRI data. PLoS Comput Biol.

[44] Du Y, Goddi A, Bortolotto C, Shen Y, Dell'Era A, Calliada F, Zhu L (2020). Wall shear stress measurements based on ultrasound vector flow imaging: theoretical studies and clinical examples. J Ultrasound Med.

[45] Gates PE, Gurung A, Mazzaro L (2018). Measurement of wall shear stress exerted by flowing blood in the human carotid artery: ultrasound Doppler velocimetry and echo particle image velocimetry. Ultrasound Med Biol.

[46] Itatani K, Sekine T, Yamagishi M (2022). Hemodynamic parameters for cardiovascular system in 4D flow MRI: mathematical definition and clinical applications. Magn Reson Med Sci.

[47] Murayama Y, Fujimura S, Suzuki T, Takao H (2019). Computational fluid dynamics as a risk assessment tool for aneurysm rupture. Neurosurg Focus.

[48] Cikes M, Sanchez-Martinez S, Claggett B (2019). Machine learning-based phenogrouping in heart failure to identify responders to cardiac resynchronization therapy. Eur J Heart Fail.

[49] Puyol-Antón E, Sidhu BS, Gould J (2022). A multimodal deep learning model for cardiac resynchronisation therapy response prediction. Med Image Anal.

[50] Wouters PC, van de Leur RR, Vessies MB (2023). Electrocardiogram-based deep learning improves outcome prediction following cardiac resynchronization therapy. Eur Heart J.

[51] Walia S, Walia S, Bhardwaj A (2026). Explainable soft-voting classifier for heart disease prediction using SHAP and LIME. Discover Computing.

[52] Larsen K, Zhao C, He Z (2026). A new method of modeling the multi-stage decision-making process of CRT using machine learning with uncertainty quantification. J Imaging Inform Med.

[53] Kovacs A, Davidovics K (2026). Regulatory and ethical challenges of cloud-based artificial intelligence in echocardiographic analysis (online). Imaging.

[54] Hu X, Zhu Y, Zhang Z (2025). Explainable artificial intelligence in echocardiography. Advanced Ultrasound in Diagnosis and Therapy.

[55] Haupt M, Maurer MH, Thomas RP (2025). Explainable artificial intelligence in radiological cardiovascular imaging - a systematic review. Diagnostics (Basel).

[56] Yang B, Qin Y, Li Y (2026). Multimodal fusion of echocardiogram images and electronic medical records for heart disease screening: retrospective algorithm development and validation study. JMIR Med Inform.

[57] Christensen M, Vukadinovic M, Yuan N, Ouyang D (2024). Vision-language foundation model for echocardiogram interpretation. Nat Med.

[58] Holste G, Oikonomou EK, Tokodi M, Kovács A, Wang Z, Khera R (2025). Complete AI-enabled echocardiography interpretation with multitask deep learning. JAMA.

